# Oolemma Receptors in Mammalian Molecular Fertilization: Function and New Methods of Study

**DOI:** 10.3389/fcell.2021.662032

**Published:** 2021-05-19

**Authors:** María Jiménez-Movilla, Julieta G. Hamze, Raquel Romar

**Affiliations:** ^1^Department of Cell Biology and Histology, Faculty of Medicine, University of Murcia, Murcia, Spain; ^2^Biomedical Research Institute of Murcia (IMIB-Arrixaca), Murcia, Spain; ^3^International Excellence Campus for Higher Education and Research “Campus Mare Nostrum”, University of Murcia, Murcia, Spain; ^4^Department of Physiology, Faculty of Veterinary Medicine, University of Murcia, Murcia, Spain

**Keywords:** fertilization, oolemma, fusion, tetraspanins, JUNO, BAI, Tim-4, Mer-TK

## Abstract

Fertilization is a key process in biology to the extent that a new individual will be born from the fusion of two cells, one of which leaves the organism in which it was produced to exert its function within a different organism. The structure and function of gametes, and main aspects of fertilization are well known. However, we have limited knowledge about the specific molecules participating in each of the steps of the fertilization process due to the transient nature of gamete interaction. Moreover, if we specifically focus in the fusion of both gametes’ membrane, we might say our molecular knowledge is practically null, despite that molecular mechanisms of cell-to-cell adhesion are well studied in somatic cells. Moreover, between both gametes, the molecular knowledge in the egg is even scarcer than in the spermatozoon for different reasons addressed in this review. Sperm-specific protein IZUMO1 and its oocyte partner, JUNO, are the first cell surface receptor pair essential for sperm–egg plasma membrane binding. Recently, thanks to gene editing tools and the development and validation of *in vitro* models, new oocyte molecules are being suggested in gamete fusion such as phosphatidylserine recognition receptors. Undoubtedly, we are in a new era for widening our comprehension on molecular fertilization. In this work, we comprehensively address the proposed molecules involved in gamete binding and fusion, from the oocyte perspective, and the new methods that are providing a better understanding of these crucial molecules.

## Introduction

To generate a new, unique, and diploid individual with the totipotent capacity to develop a species-specific organism, two highly differentiated haploid cells from each progenitor, the egg, and the sperm, must recognize themselves and bind and fuse. This event, termed fertilization, results into the generation of a zygote and involves several stages in which different molecular processes must trigger the cellular activity of the gametes. So once the sperm has reached the egg, the first contact for gamete recognition is mediated by the extracellular matrix surrounding ovulated eggs, termed zona pellucida (ZP). Initial contact ensures species-specific gamete recognition and triggers a cascade of reactions in the sperm required to penetrate through the glycoprotein coat to reach the perivitelline space and meet with the egg membrane, referred to as the oolemma. To bind and fuse, gamete’s membranes must adhere to both surfaces, and membrane proteins on each of the cells must properly recognize each other. Finally, a series of distinct membrane events must occur for two cells to fuse and merge their cytoplasmic content. All these phenomena entail the involvement of specific molecules and the activation of molecular cascades that appropriately coordinate all the cellular events that gamete recognition, binding, and fusion involve. In this mini review, we will overview these receptors with special emphasis on those located on the female gamete and participating more specifically on membrane binding and fusion.

## Oocyte Receptors in Mammalian Fertilization

Despite that cell-to-cell signaling is a fundamental biological process that has been well studied in many type of cells of a single organism (Waters and Bassler, 2005; Mathieu et al., 2019), most of the specific molecules involved in fertilization and the underlying mechanisms behind the recognition and fusion of gamete’s membranes remain largely unknown in mammals. Research in the reproduction field, and most specifically the one focused on fertilization studies based on the female gamete, has several drawbacks. For instance, egg retrieval and its use in research have bioethical implications; studies on eggs imply, in many cases, a surgical intervention or animal sacrifice; and the number of samples obtained is insufficient to carry out profound and detailed biochemical and molecular studies. Moreover, extrapolating results obtained in the fertilization process in more taxa across the tree of life, where the experimental approaches and designs are simpler, has provided interesting candidates in the fertilization process in mammals (Carlisle and Swanson, 2020). However, sequences of reproductive proteins tend to be highly divergent in closely related species and often show signatures of positive Darwinian selection (Swanson et al., 2001a,b; Swanson and Vacquier, 2002; Clark and Swanson, 2005; Clark et al., 2006; Grayson, 2015), which makes it difficult to extrapolate potential protein candidates. Moreover, in mammals, the cell-to-cell fusion process occurs among a limited number of cell types, i.e., the sperm and oocyte during fertilization, trophoblasts during placenta formation, macrophages forming giant cells, osteoclast formation, and myoblasts in the formation of myofibers and myotubes (Horsley and Pavlath, 2004). So the molecular basis of cell-to-cell fusion appears to be very specific to a small group of cell types that have also contributed to the fact that currently only a few proteins have been confirmed as essential in the mammalian oocyte fertilization.

As mentioned, recognition of gametes involves a first contact and binding of the sperm with the ZP receptors surrounding the oocyte. Although this brief review is not focused on these receptors of the first stage of fertilization but on those located at oolemma, ZP proteins will be briefly mentioned. There has been some controversy over the molecular activity of each ZP protein (ZP1–ZP4) in eutherian mammals (Wassarman, 2008), but the latest and most conclusive results using gene-deficient/modified mice indicate that the successful binding of the sperm to this extracellular egg coat is dependent on the ZP2 protein in mice and humans (Avella et al., 2013, 2014). In addition, the N-terminus of the ZP2 domain required for sperm binding regulates taxon-specific gamete recognition and is not dependent on glycosylation (Tokuhiro and Dean, 2018; Gabriela Hamze et al., 2020).

### Oolemma Receptors

Once the sperm cross the entire ZP and enter the space between the matrix and the oocyte membrane, known as the perivitelline space, they face the molecules responsible for the binding and fusion of membranes for fertilization. The first molecule in the oolemma described as essential for fertilization was CD9 (Le Naour et al., 2000; Miyado et al., 2000), a cell surface protein belonging to the tetraspanin superfamily, which participates in cell migration, adhesion, proliferation, differentiation, and signal transduction (Table 1). The tetraspanins are associated with several molecules located in the cell surface such as β1 integrins (Le Naour et al., 2000), and CD9 might be implicated in the assembly or the stabilization of these adhesion molecules (Kaji et al., 2000). To study the role of this protein in fertilization, CD9-deficient mice were generated (CD9^–/–^) and ZP-enclosed oocytes collected and inseminated *in vitro* to elucidate in which step of fertilization the failure occurred. Whereas the ZP was penetrated by wild-type sperm, it was unable to fuse with the oolemma, penetrate into ooplasm, and form pronuclei (Miyado et al., 2000). Insemination of CD9^–/–^ ZP-free oocytes was then performed observing the same pattern as with ZP-enclosed oocytes and yielding 98% fusion rates for CD9^+/+^ ZP-free oocytes and 4% for CD9^–/–^ ZP-free oocytes. When the sperm were injected into oocytes, however, similar implantation and embryo development rates were obtained from eggs derived from wild-type and CD9^–/–^ mice, thus confirming the implication of CD9 in sperm–egg fusion (Miyado et al., 2000). Later, it would be discovered that CD9 is enriched on the oocyte microvillar membrane, generating fusion-competent sites for fertilization (Jégou et al., 2011), and it is crucial for a normal shape and distribution of the membrane microvilli (Runge et al., 2007). Indeed, a recent analysis of CD9 crystal structure suggests that the highly asymmetric shape of this tetraspanin is directly involved in the clustering-induced curvature generation and its localization at high curvature regions (Umeda et al., 2020), whereas a large extracellular loop would be responsible for the critical role of CD9 in the sperm–egg fusion (Zhu et al., 2002; Umeda et al., 2020).

**TABLE 1:** Summary of oocyte’s receptors involved in mammalian fertilization known to date.

**Oocyte receptor**	**Location**	**Species**	**Sperm ligand**	**Main function**	**Assay performed**	**References**
**ZP2**	ZP	Mouse, human, pig	Unknown	Gamete recognition, sperm binding, and polyspermy prevention	Gene-edited animals, *in vitro* approaches	Baibakov et al., 2012; Avella et al., 2014; Gabriela Hamze et al., 2020
**CD9**	Oolemma	Mouse, human, cow, pig	Unknown	Sperm–egg binding/fusion, structural role	Gene-edited animals	Le Naour et al., 2000; Miyado et al., 2000; Jankovicova et al., 2019
**CD36**	Oolemma	Mouse, human	PtdSer	Sperm–egg fusion?	*In vitro* approaches	Rival et al., 2019
**CD81**	Oolemma, ZP	Mouse, human, cow, pig	Unknown	Sperm–egg binding/fusion	Gene-edited animals, *in vitro* approaches	Takahashi et al., 2001; Ohnami et al., 2012; Jankovicova et al., 2019
**Folate receptor 4 (JUNO)**	Oolemma	Mouse, human (any mammalian species?)	IZUMO1	Gamete recognition, sperm binding, and polyspermy prevention	Gene-edited animals, *in vitro* approaches	Bianchi et al., 2014; Jean et al., 2019
**BAI1**	Oolemma	Mouse, human	PtdSer	Sperm–egg fusion?	Gene-edited animals, *in vitro* approaches	Rival et al., 2019
**BAI3**	Oolemma	Mouse, human	PtdSer	Sperm–egg fusion?	*In vitro* approaches	Rival et al., 2019
**Tim-4**	Oolemma	Mouse	PtdSer	Sperm–egg fusion?	Gene-edited animals, *in vitro* approaches	Rival et al., 2019
**Mer-TK**	Oolemma	Mouse	PtdSer	Sperm–egg fusion?	Gene-edited animals, *in vitro* approaches	Rival et al., 2019

*Main function, partner protein in the sperm and type of assay performed to describe the protein main functions are shown. ZP, Zona Pellucida; BAI1, Brain-specific angiogenesis inhibitor 1; BAI3, Brain-specific angiogenesis inhibitor 3; Tim-4, T-cell immunoglobulin and mucin domain containing 4; Mer-TK, Mer tyrosine kinase; PtdSer, Phosphatidylserine.*

The oocytes express more tetraspanins in addition to CD9. For instance, CD81 has been described in mouse and human oocytes (Takahashi et al., 2001), and CD151 is also present in human eggs (Ziyyat et al., 2006). CD81-deficient female mice (CD81^–/–^) presented a 40% reduction in fertility, and *in vitro* fertilization (IVF) assays suggested that this phenotype is due to a failure in sperm–egg fusion. CD9 and CD81 might play a complementary role in gamete fusion since double knockout mice (CD9^–/–^ and CD81^–/–^) were completely infertile (Rubinstein et al., 2006; Ohnami et al., 2012). The presence of CD9 and CD81 has been recently described also in the sperm head, over the acrosome and in the plasma membrane, respectively, suggesting once more the critical role of these proteins in sperm–egg fusion (Frolikova et al., 2018). Also CD9 and CD81 have been detected in bovine and porcine oocytes’ plasma membrane, and their role in fertilization has been studied by *in vitro* approaches blocking these receptors with antibodies (Jankovicova et al., 2019). The crystal structure of CD81 has revealed that cholesterol binds CD81 within the intramembrane cholesterol-binding pocket, thus modulating conformation of the molecule (Zimmerman et al., 2016). CD81 may exist in both close and open conformations, and the equilibrium between both shifted toward the close state when cholesterol is bound, disfavoring its binding to CD19 partner (Zimmerman et al., 2016). This conformational change has also been proposed for CD9, and molecular dynamics simulation suggests that binding lipid molecules to the central cavity may disturb structural conformation, affecting binding partner affinity to EWI-2 (Umeda et al., 2020). Undoubtedly, the role of these proteins, whose activity can be modulated by their binding to oolemma components, is essential to mediate the gamete membrane binding and fusion processes for a successful fertilization.

In 2014, more than a decade after was discovered, a glycosylphosphatidylinositol (GPI)-anchored protein was identified in the oocyte as vital for fertilization (Bianchi et al., 2014). This protein encoded by the *Folr4* gene was known as Folate receptor 4 due to its putative function in folate uptake. The expression of this protein in mice CD4^+^CD25^+^ regulatory T-lymphocytes, as well as its function as a mediator of responses to dietary folate and its use in anti-tumor therapy, had been widely studied, but its presence and function in mammalian oocytes were unknown until 2014. In this study, it was demonstrated that Folr4 protein was unable to bind folate, but it was essential for fertilization, so the authors renamed this protein “JUNO,” in honor of the fertility and marriage Roman goddess (Bianchi et al., 2014). In this work, the key role of JUNO in the interaction between the sperm membrane and the oolemma was assessed by IVF assays in mice. Firstly, when IVF was performed pre-incubating for 10 min zona-free eggs with an anti-JUNO antibody, no fertilization occurred. Secondly, female JUNO-deficient mice (JUNO^–/–^) were generated, being infertile. No differences were observed in the oocyte’s morphology nor in the number of ovulated oocytes when compared with wild-type mice. However, eggs from JUNO^–/–^ mice were not fertilized after natural mating nor IVF. Moreover, ZP-free-JUNO^–/–^ oocytes were also tested in IVF, obtaining the same result. Altogether, these experiments confirmed the involvement of JUNO in sperm–egg binding. Another interesting observation from Bianchi and Wright’s study (Bianchi et al., 2014) was that JUNO seems to be involved in the block of polyspermy occurring after fertilization by protein shedding from the oolemma within 40 min after sperm penetration.

Two years after JUNO’s identification as the IZUMO1 binding partner in the sperm, several research groups revealed the tertiary structure of JUNO and JUNO–IZUMO1 complex, thus allowing the identification of the precise amino acids involved in this recognition through van der Waals interactions, suggesting that W148 of IZUMO1 interacts with L81, L82, M83, and P84 of JUNO and that W62 of JUNO interacts with R160, K161, S162, and Y163 of IZUMO1, being responsible for sperm–oocyte plasma membrane interaction (Aydin et al., 2016; Han et al., 2016; Kato et al., 2016; Ohto et al., 2016; Inoue, 2017). Besides, studies focused on knowing the precise time of JUNO expression during oocyte maturation have been performed in mice, concluding that JUNO expression starts when the oocyte reaches 13- to 22-μm diameter (Suzuki et al., 2017), coincident with the time that oocytes gain the ability to fuse with the sperm, at 20 μm (Zuccotti et al., 1994). It was also found that JUNO was expressed at the prophase I, germinal vesicle stage; and its expression continued at similar levels until metaphase II stage, thus being accumulated in oolemma during oocyte maturation (Suzuki et al., 2017). Also in humans, a recent study confirmed that JUNO is involved in human gamete recognition via interaction with human IZUMO1 and that its expression progressively increases during oocyte maturation, starting at prophase I and significantly increasing through metaphase I and II stages (Jean et al., 2019). In order to determine how CD9 and JUNO cooperate, a recent work performed a detailed analysis of the area where the sperm–egg fusion occurs, revealing that whereas CD9 is still fully present, JUNO disappears from the oocyte’s surface in concentric circles, suggesting that the molecular dynamics of CD9 and JUNO are differentially regulated (Inoue et al., 2020). Moreover, JUNO localization was abnormal in oocytes obtained from CD9^–/–^ mice since JUNO was localized in the cortical actin cap region lacking microvilli, whereas it was nearly absent from the cortical actin cap in the wild-type oocytes, suggesting that CD9 may play a crucial role in the molecular compartmentalization of GPI-anchored proteins (Inoue et al., 2020).

To date, only the few proteins in the oolemma mentioned above have been demonstrated to be crucial for gamete binding, thus contributing to fertilization process. Almost all of them have been described thanks to the use of gene editing technologies by generating gene-deficient/modified mice. However, we could define the criterion of “essentiality” too strict to be able to address the molecules that may be involved in fertilization process mainly due to a redundant effect of the participating molecular cascades and that some candidates may have a complex and fundamental activation in other cellular processes so that its deficiency makes the organism unviable. This may be the case of the proteins involved in the membrane fusion process, where no candidate with a specific function in fusion has been described in the oocyte and that is totally essential (Bianchi and Wright, 2020). Indeed, recently, it has been defined that the role of a phosphatidylserine (PtdSer) is exposed on the head region of the sperm as a key player in sperm–egg fusion (Rival et al., 2019). Interestingly, the authors in this study have found similarities in gene expression pattern between proteins involved in cell fusion on skeletal myoblasts and egg, so that several PtdSer receptors described in myoblast have been detected in oocytes, i.e., CD36, brain-specific angiogenesis inhibitor 1 (BAI1), brain-specific angiogenesis inhibitor 3 (BAI3), T-cell immunoglobulin and mucin domain containing 4 (Tim-4), and Mer tyrosine kinase (Mer-TK). Using antibodies that bind to PtdSer (BAI1/BAI3- and CD36) or to PtdSer-binding domain (Annexin V) and single- or double-knockout mice (for Tim-4, BAI1, and Mer-TK), this study concludes that even with the considerable redundancy among the PtdSer receptors, they contribute to sperm–egg fusion.

### New Methods to Study Mammalian Molecular Fertilization

Despite extensive research, the identity and presence of oolemma receptors and fusogens remain ambiguous, and our comprehension of the exact mechanisms mediating gamete binding and fusion is incomplete due to the transient nature of the binding event and the highly orchestrated and dynamic fusion mechanism. Therefore, the concomitant use of different methods can better inform us about the role of a specific molecule involved in fertilization. The methods used up to now can be classified into *in vivo* models, i.e., gene-edited animals, and *in vitro* models, i.e., solubilized, purified, and recombinant proteins (Caballero-Campo et al., 2006; Chiu et al., 2008; Bhandari et al., 2010), and antibodies against the protein of interest (Jankovicova et al., 2019). Undoubtedly, gene-edited mice have been of the outmost importance to reveal new molecules involved on the different fertilization stages. However, this technology is expensive, time-consuming, and not easily transferrable to livestock species, where it is not as much developed as in the murine model. So alternative strategies for studying molecular fertilization have been used. We anticipate that numerous candidates may emerge thanks to application of high-sensibility new screening strategies such as low-input proteomics (Virant-Klun et al., 2016), large-scale mutagenesis screens, and protein-homology analysis from diverse taxa. However, efficient and low-time-consuming experiments should be applied to optimize the experimental conditions to recapitulate gamete binding and fusion. The most recent technology applied to screen the binding and fusion of gametes is here reviewed.

#### Binding Studies: AVEXIS and Protein-Coated Beads

**FIGURE 1:**
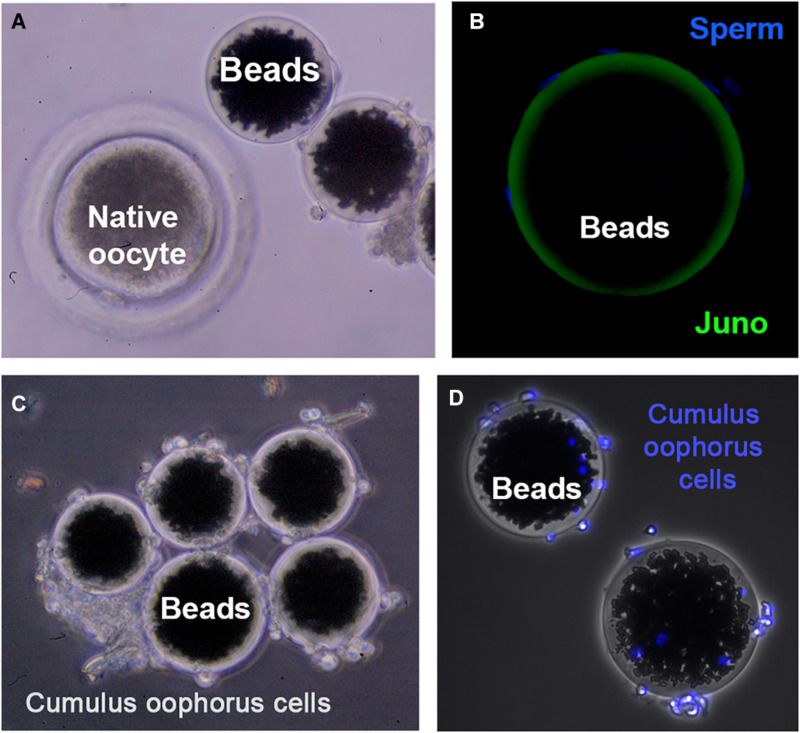
Three-dimensional (3D) model for studying gamete interaction. **(A)** Protein-coated beads and native (porcine) denuded oocyte observed under a stereomicroscope. **(B)** Confocal microscopy images of beads conjugated to recombinant JUNO protein incubated with anti-FLAG antibody. Uniform coating of bead surface and sperm bound to the 3D model after a 2-h-period co-incubation with sperm observed. Sperm DNA is stained with Hoechst (blue). **(C)** 3D model enrichment by binding cumulus cells observed under stereomicroscope. **(D)** Fluorescence microscopy images showing cumulus cells stained with Hoechst (blue) tightly adhered to the protein-coated beads.

Direct interaction between JUNO (in the egg) and IZUMO1 (in the sperm) was showed using AVEXIS (AVidity-based EXtracellular Interaction Screen), an ELISA-style assay designed to identify low-affinity interactions between extracellular proteins (Bushell et al., 2008). Direct protein interactions can be detected with the bait protein immobilized in microtiter plates or glass slides and can be scaled to systematically test many thousands of interactions in parallel (Wright and Bianchi, 2016). The advantages of this approach are based on two principles: (i) the increase of the overall binding activity by the use a short peptide sequence, which has the intrinsic property of forming pentamers, thereby increasing the local concentration of the expressed ectodomains, thus permitting their detection; and (ii) the ectodomains are expressed in mammalian cells to ensure correct folding of the ectodomain region. This assay also helped to confirm that the IZUMO–JUMO interaction is conserved within mammals due to the existence of *IZUMO1* and *JUNO* orthologs in all sequenced mammalian genomes. Since gamete binding requires a multiplicity of receptor–ligand interactions, these flat assays are an interesting and very useful tool to screen protein–protein interactions. However, physiological conditions are not fully recapitulated, since fertilizing sperm interact with a spherical egg, which is surrounded by a multilayer of *cumulus oophorus* cells at the fertilization time. Therefore, the use of three-dimensional (3D) complementary approaches is needed to expand our knowledge on the molecules involved in fertilization. In this sense, agarose or sepharose beads coated with the protein of interest are an extremely valuable tool to recreate the oocyte’s size, shape, and surface. For instance, in mice, agarose beads coated with recombinant ZP2 peptide have been used to select human sperm *in vitro*, decoy mouse sperm *in vivo*, and provide reversible contraception (Avella et al., 2016). Our group scaled this 3D model by using sepharose beads coated with an internal layer of recombinant ZP glycoproteins and a second external layer of cumulus cells (Hamze et al., 2019; Figure 1). This more physiological 3D model might help to address some of the limitations derived by the scarcity of eggs in some species, the ethical concerns in harvesting eggs, and the high cost of producing gene-edited animals. Thanks to the ability of the 3D model to decoy the sperm, the protein profile of the fertilizing bound sperm can be studied in a simple way, and in the near future, it might be useful as a new tool to select the sperm with high fertilizing capacity, to unmask subfertile animals in livestock breeding centers, or to perform toxicological studies (Hamze et al., 2020a). In fact, we have recently shown that the sperm binding to JUNO protein-coated beads is directly related to *in vitro* sperm penetrability becoming a potential tool to predict bull sperm fertilizing ability (Hamze et al., 2020b). In addition, ZP2-coated beads improve the efficiency of porcine *IVF*, increasing the rate of monospermic fertilization (Gabriela Hamze et al., 2020).

#### Fusion Studies: COS-7 Cells and C2C12 Myoblasts

Gamete membrane fusion is the capstone of fertilization; but unlike binding, fusion studies are more complex since the evidence of cell-to-cell fusion is key to verify the role of possible candidates, and it is essential in the use of cellular systems. In this sense, membrane fusion evidence has been performed by the observation of syncytia formation induced by ectopical expression of recombinant protein candidates in insect or mammalian cells such as COS-7 cells (adherent monkey kidney fibroblasts) (Inoue et al., 2015) and C2C12 mouse myoblasts (Rival et al., 2019). In the study by Inoue et al. (2015), IZUMO1 and JUNO were ectopically expressed in COS-7 cells; and whereas cell adhesion occurred, no cell fusion was recorded, thus indicating that the IZUMO1–JUNO interaction solely ensures membrane gamete adhesion but is not sufficient to induce membrane gamete fusion. More recently, a new model for cell-to-cell fusion with potential application in sperm–egg fusion has been proposed by the incubation of caudal epididymal mouse sperm with C2C12 myoblasts (Rival et al., 2019). In this study, the fusion of the sperm with skeletal myoblasts required PtdSer on the sperm and BAI1/3, ELMO2, and RAC1 in myoblasts. A series of experiments led to conclude that PtdSer on the sperm and the PtdSer recognition receptors on the oocyte (BAI1/3, CD36, Tim-4, and Mer-TK) can promote gamete membrane fusion. Moreover, the authors suggest that this fusion would occur via the ELMO–RAC1 signaling pathway since sperm entry was disturbed in oocytes lacking the cytoplasmic ELMO1 or having a functional disruption of RAC1.

The current hypothesis for sperm–egg binding and fusion extracted from the above studies is that the IZUMO1–JUNO interaction provides the initial strong gamete binding, and this would be followed by the interplay between PtdSer on the sperm and the PtdSer receptors on oocyte. At this point, the BAI1/3–ELMO–RAC1 module, along with CD9 and other oocyte molecules linked to fertilization, would help in achieving a complete and stable gamete fusion. Undoubtedly, we are in a new era for widening our comprehension on molecular fertilization, and in the near future, these new study methods will shed light on the identification and function of new oocyte receptors.

## Conclusion

Our current knowledge on the identity of specific molecules involved in different stages of fertilization in mammals is very limited despite that the cell-to-cell communication process is well studied in other cell types. Nowadays, just a few of these molecules are known in gametes, being JUNO (oocyte) and IZUMO1 (sperm), the only pair of proteins described. Gene-edited animals have provided useful information about the different receptors and their function, but emerging approaches are casting new potential molecules to study in the near future.

## Author Contributions

RR and MJ-M conceived and drafted the manuscript. MJ-M, JH, and RR wrote the manuscript, discussed its content, and revised the work. All authors approved the final submitted version.

## Conflict of Interest

The authors declare that the research was conducted in the absence of any commercial or financial relationships that could be construed as a potential conflict of interest.
